# SLC transporters ASCT2, B^0^AT1‐like, y^+^LAT1, and LAT4‐like associate with methionine electrogenic and radio‐isotope flux kinetics in rainbow trout intestine

**DOI:** 10.14814/phy2.14274

**Published:** 2019-11-08

**Authors:** Van P. T. H. To, Karthik Masagounder, Matthew E. Loewen

**Affiliations:** ^1^ Veterinary Biomedical Sciences University of Saskatchewan Saskatoon Saskatchewan Canada; ^2^ Evonik Nutrition & Care GmbH Rodenbacher Chaussee Hanau Germany

**Keywords:** Electrogenic, intestine, methionine, radioisotope flux, rainbow trout, transport

## Abstract

Methionine (Met) is an important building block and metabolite for protein biosynthesis. However, the mechanism behind its absorption in the fish gut has not been elucidated. Here, we describe the fundamental properties of Met transport along trout gut at *µ*mol/L and mmol/L concentration. Both electrogenic and unidirectional DL‐[^14^C]Met flux were employed to characterize Met transporters in Ussing chambers. Exploiting the differences in gene expression between diploid (2N) and triploid (3N) and intestinal segment as tools, allowed the association between gene and methionine transport. Specifically, three intestinal segments including pyloric caeca (PC), midgut (MG), and hindgut (HG) were assessed. Results at 0–150 *µ*mol/L concentration demonstrated that the DL‐Met was most likely transported by apical transporter ASCT2 (SLC1A5) and recycled by basolateral transporter y^+^LAT1 (SLC7A7) due to five lines of observation: (1) lack of Na^+^‐independent kinetics, (2) low expression of B^0^AT2‐like gene, (3) Na^+^‐dependent, high‐affinity (*K*_m_, *µ*mol/L ranges) kinetics in DL‐[^14^C]Met flux, (4) association mRNA expression with the high‐affinity kinetics and (5) electrogenic currents induced by Met. Results at 0.2–20 mmol/L concentration suggested that the DL‐Met transport is likely transported by B^0^AT1‐like (SLC6A19‐like) based on gene expression, Na^+^‐dependence and low‐affinity kinetics (*K*_m_, mmol/L ranges). Similarly, genomic and gene expression analysis suggest that the basolateral exit of methionine was primarily through LAT4‐like transporter (SLC43A2‐like). Conclusively, DL‐Met uptake in trout gut was most likely governed by Na^+^‐dependent apical transporters ASCT2 and B^0^AT1‐like and released through basolateral LAT4‐like, with some recycling through y^+^LAT1. A comparatively simpler model than that previously described in mammals.

## Introduction

Methionine (Met) is a sulfur‐containing essential amino acid (EAA) that plays an important role in numerous metabolic processes. Three primary functions of this EAA are protein biosynthesis, methyl donor, and precursor of cysteine synthesis (Bin et al. 2017; Métayer‐Coustard et al. 2010; Tesseraud et al. 2009). Influences of Met‐deficient diets on feed consumption, growth performance, immune response, mRNA translation efficiency, oxidative status, and protein turnover have been observed in a variety of fish species (Belghit et al. 2014; Espe et al. 2014; Kuang et al. 2012; Michelato et al. 2018; Séité et al. 2018; Tulli et al. 2010). The intestinal tract is a critical site for animal nutrient uptakes. Compared to mammals, considerably less information is available on the nutrient absorptive mechanisms in aquatic species. First studies on AAs absorption in fish include goldfish *Carassius auratus* (Mepham and Smith 1966), white grunt *Haemulon plumieri* (Smith 1969), rainbow trout *Salmo gairdneri* (Ingham and Arme 1977), killifish *Fundulus heteroclitus* (Miller and Kinter 1979), European yellow eel *Anguilla anguilla* (Maffia et al. 1990), and sea bass *Dicentrarchus labrax* (Balocco et al. 1993). Most of these studies have demonstrated that the Na^+^ electrochemical gradient is the driving force for the absorption of AAs.

AA absorption in the intestine is a complex process and an individual AA is typically transported by multiple transporters. AA transporter systems belong to solute carrier (SLC) gene superfamily and can be grouped into some categories such as neutral, basic and acidic systems. Being a neutral AA, Met transports have been described in both Na^+^‐dependent (system A, system ASC, system B^0^, aka NBB, system IMINO and system y^+^L) and Na^+^‐independent pathways (system L, system b^0,+^‐like, system y^+^‐like) (Bröer 2008; Kobayashi et al. 2012; Malo 1991; Munck et al. 2000; Soriano‐García et al. 1998). As mentioned earlier, current knowledge of how nutrients absorbed in the fish intestine in comparison to mammals is very limited due to the diversity of the living environment and gut anatomy of aquatic species.

Here, we exploit the differences in the absorption kinetics of DL‐Met and transport gene expression between diploid and triploid rainbow trout as tools to determine the mechanism of Met absorption in fish gut. It should be noted that the intention of this study was not to give mechanism to the controversial difference in growth rate between ploidy (McGeachy et al. 1995; O’Flynn et al. 1997; Oppedal et al. 2003). Met absorption kinetics were successfully studied in the presence and absence of sodium at *µ*mol/L and mmol/L DL‐Met concentration gradients created by increasing sequential concentration of Met. Trout gut demonstrated a Na^+^‐dependent, high‐affinity kinetics at 0–150 *µ*mol/L DL‐Met concentration and a Na^+^‐dependent, low‐affinity kinetics at 0.2–20 mmol/L DL‐Met concentration. Genome and gene expression analysis indicated that ASCT2 (SLC1A5) and B^0^AT1‐like (SLC6A19‐like) were possible candidates responsible for the apical Met absorption at *µ*mol/L and mmol/L concentration gradients respectively. Whereas basolateral transporters y^+^LAT1 (SLC7A7) was associated with electrogenic recycling and LAT4‐like (SLC43A2‐like) was associated with Met exit.

## Materials and Methods

### Genomic analysis to identify methionine transporter genes

All Met transporter candidates that previously described in mammalian and aquatic species were first listed. The list included 18 transporter candidates: SNAT‐1,2,4 (SLC38A‐1,2,4), ASCT2 (SLC1A5), rBAT/b^0,+^AT (SLC3A1/SLC7A9), IMINO (SLC6A20), 4F2hc/LAT1 (SLC3A2/SLC7A5), 4F2hc/LAT2 (SLC3A2/SLC7A8), LAT3 (SLC43A1), LAT4 (SLC43A2), 4F2hc/y^+^LAT1 (SLC3A2/SLC7A7), 4F2hc/y^+^LAT2 (SLC3A2/SLC7A6), ATB^0,+^ (SLC6A14), B^0^AT1 (SLC6A19), B^0^AT2 (SLC6A15), CAT‐1,2,3 (SLC7A‐1,2,3). Zebrafish sequences were initially used as reference to identify sequences of candidate genes in trout. However, several listed genes were not available in zebrafish and the blast analysis resulted in poor identification compared to similar process employed when using human transporters sequences. Therefore, the nucleotides of 18 human transporters were used to blast against the rainbow trout genome to identify similar mRNA sequences in trout. The match between human and trout sequences was justified based on the e‐values less than 10^^‐15^ which were accepted. The results were double checked in NCBI assembly software using gene name search. Subsequently, available trout mRNA sequences were retrieved from NCBI website (http://www.ncbi.nlm.nih.gov/) and aligned using CLUSTAL W and MEGA7 software (https://www.megasoftware.net/) to create a phylogenetic tree.

### Fish source & husbandry

Diploid and triploid rainbow trout (*Oncorhynchus mykiss*) were purchased from B&B Freshwater Fish Farm (Gunton, Manitoba, Canada) and Wild West Steelhead hatchery (Lucky Lake, Saskatchewan, Canada) respectively. Fish were housed in an indoor recirculating system which included 120 L fiberglass tanks connected to a sump tank and a biofilter. Each tank was oxygenated using air stones to maintain the oxygen above 6 mg/L, and the temperature was kept at 11–12°C throughout the experiments. Fish was fed twice daily (2–3% body weight) with commercial feed containing 45% Crude Protein and 16% Lipid manufactured by EWOS Canada Limited (Surrey, British Columbia, Canada).

### Tissue collection

Healthy fish (50–150 g) were selected and transferred to processing building for gut dissection. Fish was euthanized by blunt trauma. The intestine was collected immediately after dissection, opened and rinsed carefully with teleost buffer pH 7.7, containing (in mmol/L): 118 NaCl, 2.9 KCl, 2.0 CaCl_2_·2H_2_O, 1.0 MgSO_4_·7H_2_O, 0.1 NaH_2._PO_4·_H_2_O, 2.5 Na_2_HPO_4_, 1.9 NaHCO_3,_ and 5.6 Glucose, which was adopted from the research of Small and co‐authors (Small et al. 1990). Each figure *n* represents a biological replicate of an individual fish intestinal segment. Feces and uneaten feed were removed from luminal content. Pyloric caeca, midgut, and hindgut were collected. Three segments were visually distinct from each other, which were described by Burnstock (1959). Specifically, the pyloric ceca region was located directly behind the stomach. Midgut and hindgut were located about 2 inches and 5–6 inches from the stomach respectively (Subramaniam et al. 2019).

### RNA extraction, cDNA synthesis, and quantifying gene expression using quantitative real‐time PCR (RT‐ qPCR)

About 1 mg samples of PC, MG, and HG were obtained from fish dissection and stored in RNA*later* ^®^ RNA Stabilization Solution (Fisher Scientific) at −80°C for later use of gene expression. Each figure *n* represents a biological replicate of an individual fish intestinal segment. Total RNA was extracted using Trizol (Thermo Fisher Scientific) according to the manufacturer’s instruction. RNA quality and quantity were determined with Nano‐Drop spectrophotometer (Fisher Scientific). cDNA synthesis via reverse transcription was performed using qScript cDNA Synthesis Kit (Quanta BioSciences) for 5 min at 25°C, 30 min at 42°C, and 5 min at 85°C. To evaluate PCR efficiency, cDNA templates from biological samples were diluted to create dilution standard curves. Amplification efficiency of qPCR between 90 and 100% was considered acceptable. PCR products were purified with QIAquick kit (Quiagen), sequenced and BLAST searched before proceeding to RT‐qPCR. RT‐qPCR was performed using PerfeCTa^®^ SYBR^®^ Green SuperMix (Quanta BioSciences); initiated at 95°C for 3 min, followed by 40 cycles of 95°C for 10 sec, 59°C for 10 sec and 72°C for 30 sec, using Bio‐Rad T100 Thermal Cycler (Bio‐Rad). Elongation factor alpha 1 (EF*α*1) was used as a reference to normalize mRNA expression of genes that were predicted to participate in Met transport. Primer sequences along with EF*α*1 used for RT‐qPCR were listed in Table 1.

**Table 1:** Rainbow trout (*Oncorhynchus mykiss*) primer sequences used for RT‐qPCR.

SLC^*^	System (Gene name)	Location	Forward (5′ – 3′)	Reverse (5′ – 3′)	Gene Bank Accession #
SLC1A5	ASCT (ASCT2)	AM	AAA GAG TCG GTC ATG TAG AG	GAG AGA AGA CAC AAG GAG AG	XM021587427.1
SLC3A1	b^0,+^ (rBAT)	AM	AGG CCG ATA CAG GTT TAT G	CCC AGT TCC AGT CAG ATT AG	XM021576370.1
SLC7A5	L (LAT1)	BM	TGG TCT GTT TGC CTA TGG	GTG AAG TAG GCC AGG TTA G	XM021568487.1
SLC43A1	L (LAT3)	AM?	CTG TTG CCT GGA TAC CTA TT	TAT GCT AGA CCG TTG CTA TG	XM021583981.1
SLC43A2‐like	L (LAT4‐like)	BM	GAC GGA CGG AGA TTT GTT	GAG AGA GAG AGA GAG AGA GAG	XM021582086.1
SLC7A7	y^+^L (y^+^LAT1)	BM	GAG GAC TCA ACG CTT CTA TC	CAA CAC ACA GGT AGA CCA A	XM021614955.1
SLC6A19‐like	B^0^ (B^0^AT1‐like)	AM	GGT CCA TCC TGT TCT TCA T	TGA CAC CAG ACA GAC AAT AC	XM021562073.1
SLC6A15‐like	B^0^ (B^0^AT2‐like)	AM	TCT ACT TCT CCC AGT CCT T	GGA GTC AGA GAT GTT CAG AG	XM021604100.1
SLC6A14	B^0,+^ (ATB^0,+^)	AM	TGG AGT GAC TGT TTC TAC TG	CTG GGA TGC TGA TGA TGT	XM021610363.1
SLC7A3‐like	y^+^ (CAT3‐like)	AM?	GTT TAC TGG GCT CAA TGT TC	ATC AGG GCT GCT ACA ATA C	XM021561172.1
SLC3A2	4F2hc	/	GGA TCT GAC TCC CTA CTA TCT	CCC AAA GAG ACG GAA CTA C	XM021591192.1
Housekeeping	EF*α*1		AGC GAG CTC AAG AAG AAG	GAC CAA GAG GAG GGT ATT C	NM001124339.1

SLC*, Solute carrier; AM, Apical membrane; BM, Basolateral membrane.

### Ussing chamber technique

EasyMount Ussing Chambers System model (Physiologic Instruments Inc., San Diego, CA) was used in this study. The Ussing chamber technique was adapted and earlier described in previous works (Loewen et al. 2004; Subramaniam et al. 2019). In brief, three intestinal segments (PC, MG, and HG) were mounted as flat sheets on metal pins of the inserts with the exposure of a surface area of 0.3 cm^2^. Another insert was put on the top like a sandwich which was placed into the middle of the chamber and secured with thumbwheel screws. Each side of the chamber reservoirs contained 5 mL fresh teleost saline buffer and oxygenated continuously with 1% CO_2_ and 99% O_2_ through needle valves. The buffer was maintained at similar range with fish housing temperature (12°C) throughout the experiment by a circulating water jacket connected with a heater.

### Flux transport and electrogenic studies in Ussing Chamber

#### ^14^C radiolabeled Met flux studies

The teleost buffer was prepared freshly to initiate Na^+^‐dependent experiments. Meanwhile, sodium was iso‐osmotically replaced with potassium to perform Na^+^‐independent experiments. Prior to adding radioactive isotopes, blank samples were taken to ensure that the chambers were not contaminated with isotope from previous use. 0.5 *µ*Ci of DL‐[^14^C]Met with the specific activity of 55 mCi mmol^−1^ (American Radiolabel Chemicals) was added to the apical compartment as tracers. 0.5 *µ*Ci of [^3^H]‐Inulin with the specific activity of 9.25 MBq/0.5 mCi (PerkinElmer) was used to analyze and determine the viability of the tissues afterward along with resistances. Tissues were excluded if significant inulin flux or a drop in resistance of the tissues was noted. Tissues were allowed to equilibrate for 60 min. After equilibration time, unlabeled substrate DL‐Met was added to the apical compartment, and Mannitol was added to the basolateral compartment with the same concentration to maintain an equal osmolarity. Two sets of experiments were separately studied with two levels of substrate concentration gradient: 0–150 *µ*mol/L (21 increasing sequential concentration) and 0.2–20 mmol/L (19 increasing sequential concentration). Each level included Na^+^‐dependent and Na^+^‐independent experiments. Each data increment was about 10 min long. 500 *µ*L samples were taken from “cold side” mixed with 4 mL‐UltimaGold cocktail solution (PerkinElmer) and counted using the Scintillation Counter (Beckman Coulter).

The unidirectional fluxes rates were calculated from the appearance of radiotracer on the “cold side” (aka receiver chamber or sink chamber) and specific activity in the “hot side” (aka donor chamber or source chamber) using equation described by Schultz and Zalusky to determine *J*_ms_ (Schultz and Zalusky 1964). J_ms_ was then used to determine kinetic parameters (*J*_max_ and *K*_m_).

$$J_{\text{ms}}=v_{s}\left(P_{s}2-cP_{s}1\right)/\left(\Delta t*P_{m}*A\right).$$

J_ms_ = unidirectional substrate flux from mucosa to serosa in *µ*mol/cm^2^·h.

*ν*_s_ = volume of bathing solution perfusing the serosal surface in cm^3^.

*P*_s_ = cpm/cm^3^ in the serosal reservoir.

*c* = correction factor for dilution.

*A* = area of tissue exposed.

Δ*t* = time interval between two samples in hour.

*P*_m_ = specific activity of the isotopes in the mucosal solution in cpm/*µ*mol.

#### Electrophysiological recording

It was well‐documented that a large number of the transporters involved in Met transports are Na^+^‐dependent. Therefore, although Met is a neutral amino acid, its transport could be indirectly measured via changes in short‐circuit current (*I*_sc_) due to the movement of ions such as Na^+^ altering electrical membrane potential. The detailed protocol was followed as manufacturer instruction and described previously in the guide of Ussing chamber technique (Clarke 2009). In brief, to measure the changes in *I*_sc_, the Ussing chamber system (Physiologic Instruments Inc., San Diego, CA) included two Ag/AgCl electrodes pairs: one voltage set and one current set measuring the short‐circuit current across the fish tissue via agar bridges. The electrodes were attached to a voltage/current clamp (Physiologic Instruments Inc., San Diego, CA). Tissues were then clamped to 0.0 mV and the resulting short‐circuit current measured by a computer in *µ*A. The electrode configuration would result in a positive short‐circuit current when a cation moves in the mucosal to serosal direction or an anion moving in the serosal to mucosal direction. The tissue was then pulsed with a constant 0.001V pulse to determine tissue resistance every 30s.

### Kinetics and statistics analysis

To find out *J*_max_ (presented in *µ*mol/cm^2^·h), *V*_max_ (presented in *µ*A/cm^2^), and *K*_m_ (presented in *µ*mol/L or mmol/L), changes in flux rate and short circuit current over all concentrations were computer fitted to nonlinear regression Michaelis–Menten equation denoted by equation (1) or (2) using GraphPad Prism version 5:

(1) $$J=\left\{\left(J_{\max}\times \left[S\right]\right)/\left(K_{m}+\left[S\right]\right)\right\}$$

(2) $$V=\left\{\left(V_{\max}\times \left[S\right]\right)/\left(K_{m}+\left[S\right]\right)\right\}$$

where *J*_max_ (*V*_max_) were the maximal flux rate (maximal current) at saturable substrate concentration, *K*_m_ was the substrate concentration that generates half *J*_max_ (*V*_max_), and [S] was the substrate concentration. Results were presented as means ± SEM. The *J*_max_ and *V*_max_ values were compared between ploidy using student’s *t*‐test. Similarly, *K*_m_ values were compared between ploidy using student’s *t*‐test. *J*_max_, *V*_max_ and *K*_m_ within intestinal segments of each ploidy were compared using one‐way ANOVA, followed by Tukey HSD to determine differences among intestinal segments (PC, MG, and HG). Similar statistical analysis methods were used to analyze RT‐qPCR data. All statistical tests were performed using SYSTAT version 13. A *P*‐value less than 0.05 was accepted as a statistically significant difference.

## Results

### Genomic and gene expression analysis of trout transporters involved in Met transport

**Figure 1:**
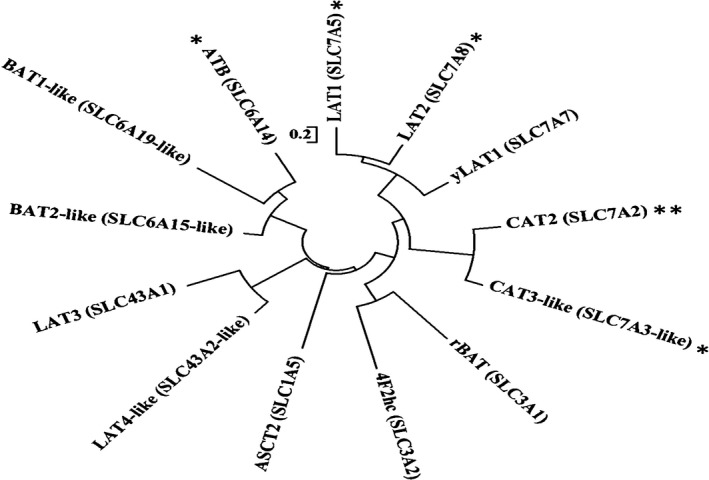
Phylogenetic tree demonstrating the diversity of Methionine‐linked transporters found in the genome of rainbow trout (*Oncorhynchus mykiss*), emphasizing complexity (both their diversity and similarity) of transporting methionine in the entire animal and gut specifically. Transporters with (*) including CAT3‐like, LAT1, LAT2, and ATB^0,+^ might have minor role in Met transport due to low mRNA expression. Whereas, mRNA expression of CAT2 (**) was not detectable. Heavy chain 4F2hc typically associates with a number of light chain including LAT1, LAT2, and y^+^LAT1, meanwhile heavy chain rBAT associates with b^0,+^AT (SLC7A9).

A genomic analysis for putative Met transports resulted in 11 mRNA sequences of Met‐linked transporters and 2 heavy subunits present in rainbow trout out of the 18 known transporters (Bröer 2008; Mastrototaro et al. 2016). Although genes including y^+^LAT2 (SLC7A6), CAT1 (SLC7A1), IMINO (SLC6A20), b^0,+^AT (SLC7A9), and SNAT‐1,2,4 (SLC38A‐1,2,4) were previously reported to have Met transport function in mammalian and poultry (Bröer 2008; Chen et al. 1994; Mackenzie et al. 2003; Mastrototaro et al. 2016; Nickel et al. 2009; Soriano‐García et al. 1998; Yao et al. 2000; Zhang et al. 2015), genomic analysis did not detect these genes. Figure 1 illustrates multiple transporters found, emphasizing complexity (both their diversity and similarity) of transporting methionine in the entire animal and gut specifically.

Among 11 genes and two heavy subunits found in the genome, RT‐qPCR was performed in the three intestinal segments of 3N and 2N trout to identify which genes were present and potentially responsible for Met transport along the trout gut. Despite being present in the trout genome, RT‐qPCR did not detect CAT2 (SLC7A2) in the intestine; while CAT3‐like (SLC7A3‐like), LAT1 (SLC7A5), LAT2 (SLC7A8), and ATB^0,+^ (SCL6A14) had exceptionally low mRNA expression. More specifically the relative expression of these genes to EF*α*1 was less than 0.005, suggesting a minor or insignificant role of these transporters in gut methionine transport of rainbow trout. However, RT‐qPCR analysis had identified six candidate genes that might have direct contribution to Met transport and regulation in the intestine of rainbow trout including ASCT2 (SLC1A5), B^0^AT1‐like (SLC6A19‐like), B^0^AT2‐like (SLC6A15‐like), y^+^LAT1 (SLC7A7), LAT3 (SLC43A1), and LAT4‐like (SLC43A2‐like) along with the heavy subunits rBAT (SLC3A1) and 4F2hc (SLC3A2) which are typically required for some transporters to be functional.

More specifically, RT‐qPCR results showed that there was the mRNA expression of both high‐affinity transporters (ASCT2, y^+^LAT1, B^0^AT2‐like) and low‐affinity transporters (B^0^AT1‐like, LAT3, LAT4‐like). Details of the gene expression are presented along with kinetic analysis in the following sections. Due to the two transporter populations (high affinity and low affinity transports) we performed flux experiments in both micromolar (0–150 *µ*mol/L) and millimolar (0.2–20 mmol/L) substrate concentration ranges to characterize the kinetics associated with these genes. Furthermore, these ranges were chosen due to relevance in aquaculture where plant‐based diet can have exceptionally low methionine and supplementation can increase levels to mmol/L concentration.

### Transport of DL‐Met at micromolar concentration

#### ^14^C radiolabeled met flux

**Figure 2:**
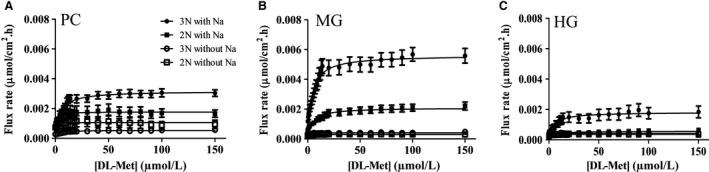
Transport of DL‐Met at micromolar (*µ*mol/L) concentration. Michaelis‐Menten plots for the DL‐[ ^14^C]Met flux assays in the presence of Na^+^ (*n* = 19–25) and absence of Na^+^ (*n* = 17–20) in (A) pyloric caeca (PC), (B) midgut (MG) and (C) hindgut (HG) of triploid (●3N, ○) and diploid (■ 2N, □2N). Experiments were carried out with DL‐Met gradient from 0 to 150 *µ*mol/L (21 increasing sequential concentration). Each data point was expressed as mean ± SEM.

DL‐[^14^C]Met flux rate was performed in the presence or absence of Na^+^ at a range of substrate concentration of 0–150 *µ*mol/L. In Na^+^ buffer, the flux rate of DL‐[^14^C]Met in all three intestinal segments exhibited saturable kinetics with increasing concentration of DL‐Met (Fig. 2). Segmental comparison demonstrated that the flux rate (*J*_max_) was signficantly higher in the PC and MG than in the HG. Table 2 showed that this phenomenon was observed in both types of ploidy (*P* < 0.0001). Moreover, ploidy comparison demonstrated that DL‐[^14^C]Met flux rate in the gut of 3N trout (0.003, 0.006, and 0.002 *µ*mol/cm^2^·h in PC, MG, and HG respectively) was statistically higher than that of 2N trout (0.0019, 0.0021, and 0.0006 *µ*mol/cm^2^·h in PC, MG, and HG respectively). The analysis of kinetic constants revealed that the high affinity for DL‐[^14^C]Met was identical (*K*_m_ values between 4 and 5 *µ*mol/L) in the entire intestinal tract regardless of type of ploidy or segment. On the contrary, the flux rate of DL‐[^14^C]Met was not a function of substrate concentration when the assays were carried out in Na^+^‐free buffer. Data from sodium free experiments could not be fitting to Michaelis‐Menten equation nor linear regression (negative or poor R^2^ values). These observations indicated that DL‐Met transport at micromolar concentrations was strictly dependent on the existence of a Na^+^‐dependent, high‐affinity transporter.

**Table 2:** Transport of DL‐Met at micromolar (*µ*mol/L) concentration.

Intestinal segments	*J*_max _(*µ*mol/cm^2^·h)			*K*_m_ (*µ*mol/L)		
	Triploid	Diploid	*J*_max_ between triploid vs. diploid (*P*‐value)	Triploid	Diploid	*K*_m_ between triploid vs. diploid (*P*‐value)
PC	0.003 ± 0.0003	0.0019 ± 0.0003	0.004^*^	5.48 ± 0.65	4.01 ± 0.86	0.45
MG	0.006 ± 0.0005	0.0021 ± 0.0003	<0.0001^*^	4.64 ± 0.41	4.71 ± 0.71	0.97
HG	0.002 ± 0.0004	0.0006 ± 0.00001	0.007^*^	3.93 ± 0.84	4.33 ± 0.95	0.98

*J*_max_ and *K*_m_ values generated by DL‐ [ 14C]Met isotope flux along the gastrointestinal tract of rainbow trout in the Na^+^ buffer, substrate DL‐Met gradient from 0 to 150 *µ*mol/L. Values were expressed as mean ± SEM (*n* = 19–25). Asterisks represent significant difference in *J*_max_ between ploidies (Student’s *t*‐test, *P < 0.05).

#### Gene expression of high‐affinity transporters candidates affirm high‐ affinity transport kinetics at micromolar concentrations

**Figure 3:**
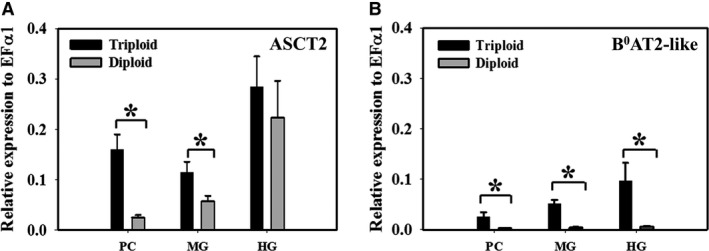
Expression of sodium dependent high‐affinity (*µ*mol/L) apical transporters. (A) Relative mRNA expression of Na^+^‐dependent high‐affinity apical transporter ASCT2 (SLC1A5) and (B) Relative mRNA expression of Na^+^‐dependent high‐affinity apical transporter B^0^AT2‐like (SLC6A15‐like) in pyloric caeca (PC), midgut (MG), and hindgut (HG) of triploid and diploid rainbow trout using RT‐qPCR. Housekeeping gene EF*α*1 was used for normalization of mRNA abundance data. Values were expressed as means ± SEM (*n* = 8–10). Asterisks represent significant differences between ploidy (Student’s *t*‐test, *P* < 0.05).

Among genes identified, ASCT2 (SLC1A5) and B^0^AT2‐like (SLC6A15‐like) were Na^+^‐dependent, high‐affinity transporters located in the apical membrane. There were statistically significant differences in gene expression between 2N and 3N trout. Figure 3A showed that mRNA expression of ASCT2 in PC (*P* = 0.005) and MG (*P* = 0.026) of triploid were significantly greater than that of diploid. This supports the greater transport rate in 3N fish at micromolar concentrations. A similar difference was observered with B^0^AT2‐like mRNA expression (Fig. 3B, *P* < 0.05 in all three intestinal segments). However, the expression of B^0^AT2‐like transporter was less than ASCT2, particularly in diploid fish.

**Figure 4:**
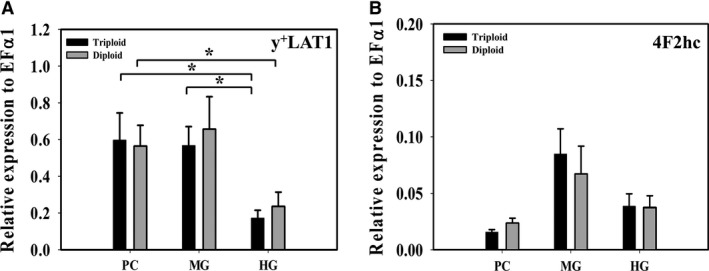
Expression of sodium dependent high‐affinity (*µ*mol/L) basolateral transporter and its heavy subunit. (A) Relative mRNA expression of Na^+^‐dependent high‐affinity basolateral transporter y^+^LAT1 (SLC7A7) and (B) Relative mRNA expression of heavy subunit 4F2hc (SLC3A2) in pyloric caeca (PC), midgut (MG), and hindgut (HG) of triploid and diploid rainbow trout using RT‐qPCR. Housekeeping gene EF*α*1 was used for normalization of mRNA abundance data. Values were expressed as means ± SEM (*n* = 8–10). Asterisks represent significant differences among intestinal segments (one‐way ANOVA, *P* < 0.05).

Additionally, y^+^LAT1 (SLC7A7), a Na^+^‐dependent, high‐affinity transporter usually localized in the basolateral membrane was found to have expression throughout the intestine (Fig. 4A). Data analysis revealed that mRNA expression of y^+^LAT1 was significantly lower in HG than in PC and MG (*P* = 0.019 in triploid, *P* = 0.037 in diploid). There was no difference in the expression of 4F2hc, a heavy subunit of y^+^LAT1 (Fig. 4B). Finally, heavy chain rBAT (SLC3A1) of Na^+^‐independent, high‐affinity apical transporter b^0,+^AT (SLC7A9) was found to have dominant expression along the trout gut, particularly in HG (data not shown). However, RT‐qPCR could not be performed for the conducting light chain b^0,+^AT due to its absence in the genome. This is consistent with our flux studies which showed no transport of Met in Na^+^‐independent experiments, suggesting that rBAT/b^0,+^AT did not directly facilitate the Met uptakes. Thus, we assumed that Met transport at the concentration 0‐150 *µ*mol/L is primarily mediated by ASCT2 and y^+^LAT1.

#### Electrophysiological recordings support the presence of apical transporter ASCT2 and basolateral transporter y^+^LAT1 functional expression

**Figure 5:**
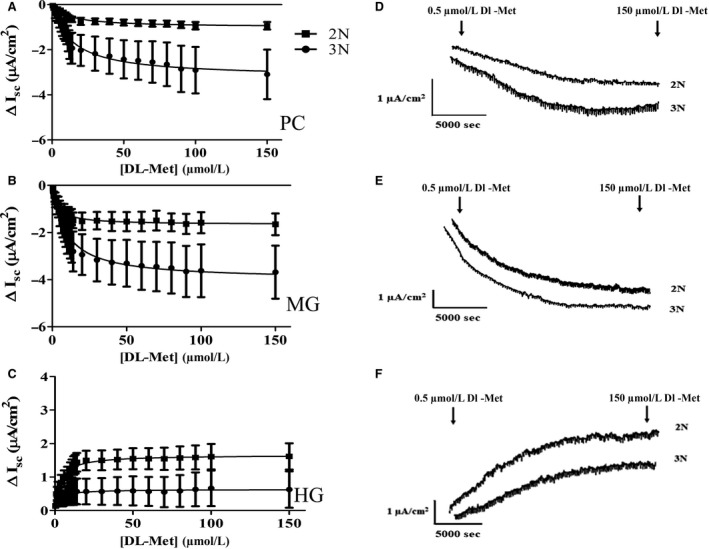
Electrogenic short‐circuit current (*I*_sc_) induced by DL‐Met. Increasing concentrations of DL‐Met in Na^+^ media induced changes in *I*_sc_ (A) in pyloric caeca (PC), (B) midgut (MG), and (C) hindgut (HG) of triploid (● 3N) and diploid (■ 2N) with segmental representative traces (D, E, and F) presented on the right side. The DL Met‐induced current was plotted as a function of the extracellular substrate concentrations using Michaelis–Menten equation. Experiments were carried out with DL‐Met gradient from 0 to 150 *µ*mol/L (21 increasing sequential concentration). Each data point was expressed as mean ± SEM (*n* = 13–18).

In the presence of sodium, DL‐Met gradient induced an *I*_sc_ change in the intestine of rainbow trout. Increasing substrate concentration from 0 to 150 *µ*mol/L resulted in a negative *I*_sc_ in the PC and MG; and a positive *I*_sc_ in the HG (Fig. 5). The changes in *I*_sc_ were found to display saturable kinetics resulting in a small *K*_m_ (micromolar ranges) regardless of segments (Table 3). Overall when comparing the effects of ploidy on the rate of short circuit current, *V*_max_ tended to be higher in 3N trout compared to *V*_max_ in 2N trout. *P*‐values were 0.012 in PC (3.87 ± 1.10 *µ*A/cm^2^ in triploid vs. 1.12 ± 0.19 *µ*A/cm^2^ in diploid) and 0.054 in MG (4.24 ± 1.03 *µ*A/cm^2^ in triploid vs. 2.94 ± 0.70 *µ*A/cm^2^ in diploid) respectively. The larger negative *I*_sc_ was associated with higher expression of ASCT2 in triploid PC and MG (Fig. 3A), likely resulting in higher basolateral Met available for y^+^LAT1 recycling causing a larger negative *V*_max_ in triploid (Fig. 5). Whereas the positive *I*_sc_ in the HG was associated with a decrease in y^+^LAT1 (Fig. 4A) and an increase in ASCT2 in both triploid and diploid (Fig. 3A). The observation of electrophysiological recordings of DL‐Met‐induced currents and associated gene expression affirmed that possibly Na^+^‐dependent, high‐affinity transporter ASCT2 and y^+^LAT1 facilitated DL‐Met uptake at the range of substrate concentration less than 150 *µ*mol/L.

**Table 3:** Electrogenic *V*_max_ and *K*_m_ values generated by DL‐Met along the gastrointestinal tract of rainbow trout in Na^+^ buffer at micromolar (*µ*mol/L) concentration.

Intestinal segments	*V*_max_ (*µ*A/cm^2^)			*K*_m_ (*µ*mol/L)		
	Triploid	Diploid	*V*_max_ between triploid vs. diploid (*P*‐value)	Triploid	Diploid	*K*_m_ between triploid VS diploid (*P*‐value)
PC	3.87 ± 1.10	1.12 ± 0.19	0.012^*^	15.5 ± 4.01	14.6 ± 3.31	0.57
MG	4.24 ± 1.30	2.94 ± 0.70	0.054	6.3 ± 1.72	6.2 ± 1.18	0.26
HG	1.94 ± 0.26	1.81 ± 0.34	0.504	13.54 ± 3.47	6.44 ± 1.11	0.052

Values were expressed as mean ± SEM (*n* = 13–18). Asterisks represent significant differences in *V*_max_ between ploidies (Student’s *t*‐test, *P < 0.05).

### Transport of DL‐Met at millimolar concentration

#### ^14^C radiolabeled Met flux results

**Figure 6:**
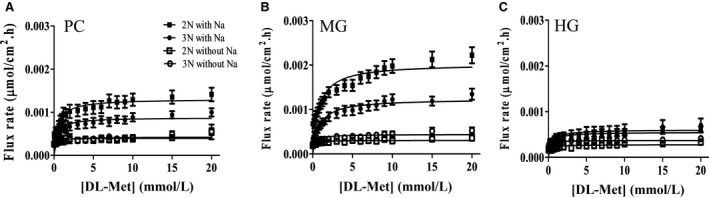
Transport of DL‐Met at millimolar (mmol/L) concentration. Michaelis–Menten plots for the DL‐[ ^14^C]Met flux assays in the presence of Na^+^ (*n* = 21–27) and absence of Na^+^ (*n* = 14–20) in (A) pyloric caeca (PC), (B) midgut (MG), and (C) hindgut (HG) of triploid (●3N,○) and diploid (■ 2N, □2N). Experiments were carried out with DL‐Met gradient from 0.2 to 20 mmol/L (19 increasing sequential concentration). Each data point was expressed as mean ± SEM.

DL‐[^14^C]Met flux rate was performed in the presence or absence of Na^+^ at a higher substrate DL‐Met concentration gradient 0.2–20 mmol/L. In Na^+^‐condition, the result demonstrated that the flux rate of DL‐[^14^C]Met at mmol/L concentration fitted Michaelis Menten kinetics, revealing another transporter that mediated Met transport at mmol/L substrate concentration. The kinetic analysis demonstrated low affinity kinetics (mmol/L ranges) (Table 4). *J*_max_ in PC and MG of 3N trout were 0.0009 ± 0.0001 and 0.0013 ± 0.0001 *µ*mol/cm^2^·h respectively. These were significantly lower than 0.0014 ± 0.0002 and 0.002 ± 0.0002 *µ*mol/cm^2^·h in PC and MG, respectively, of 2N trout (Fig. 6). These differences could be accounted for by the higher mRNA expression of B^0^AT1‐like in diploid, the significance of which is discussed below. Additionally, data could not be fitted to Michaelis‐Menten equation (negative R^2^) in Na^+^‐independent assays and reduced flux to almost zero. This indicated that DL‐Met was strictly regulated by Na^+^‐dependent transporter even at mmol/L DL‐Met concentrations.

**Table 4:** Transport of DL‐Met at millimolar (mmol/L) concentration.

Intestinal segments	*J*_max_ (*µ*mol/cm^2^·h)			*K*_m_ ( mmol/L)		
	Triploid	Diploid	*J*_max_ between triploid vs. diploid (*P*‐value)	Triploid	Diploid	*K*_m_ between triploid VS diploid (*P*‐value)
PC	0.0009 ± 0.0001	0.0014 ± 0.0002	0.035^*^	0.65 ± 0.12	0.73 ± 0.10	0.659
MG	0.0013 ± 0.0001	0.002 ± 0.0002	<0.0001^*^	1.00 ± 0.09	0.98 ± 0.13	0.897
HG	0.0006 ± 0.0001	0.0006 ± 0.00001	0.696	0.55 ± 0.09	0.67 ± 0.14	0.623

*J*_max_ and *K*_m_ values generated by DL‐ [ 14C]Met flux assays along the gastrointestinal tract of rainbow trout in the Na ^+^ buffer, substrate DL‐Met gradient from 0.2 to 20 mmol/L. Values were expressed as mean ± SEM (*n* = 21–27). Asterisks represent significant difference in *J*_max_ between ploidies (Student’s *t*‐test, *P < 0.05).

#### Gene expression of low‐affinity transporters candidates affirm low‐affinity kinetics

**Figure 7:**
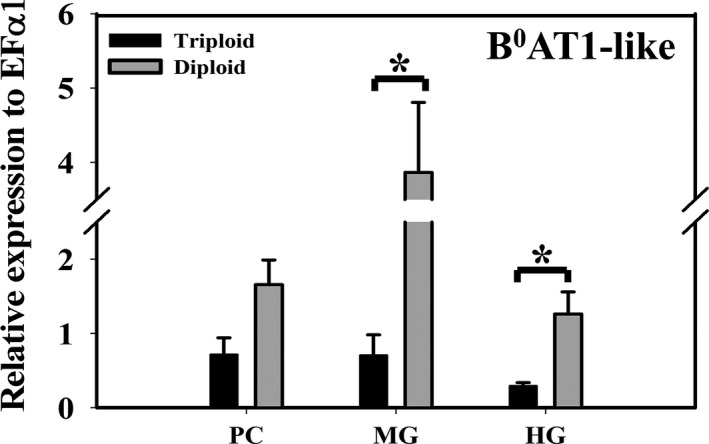
Expression of sodium dependent low‐affinity (mmol/L) apical transporter. Relative mRNA expression of Na^+^‐dependent low‐affinity apical transporter B^0^AT1‐like (SLC6A19‐like) in pyloric caeca, midgut, and hindgut of triploid and diploid rainbow trout using RT‐qPCR. Housekeeping gene EF*α*1 was used for normalization of mRNA abundance data. Values were expressed as means ± SEM (*n* = 8–10). Asterisks represent significant differences between ploidy (Student’s *t*‐test, *P* < 0.05).

**Figure 8:**
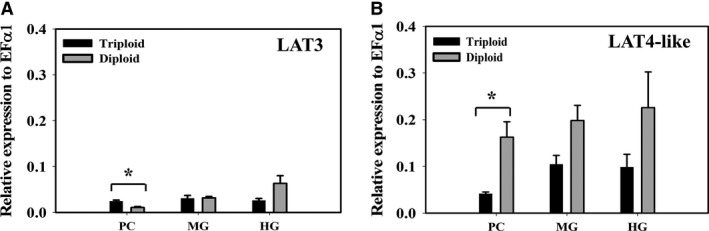
Expression of sodium independent low‐affinity (mmol/L) transporters. (A) Relative mRNA expression of Na^+^‐independent low affinity apical transporter LAT3 (SLC43A1) and (B) Relative mRNA expression of Na^+^‐independent low‐affinity basolateral transporter LAT4‐like (SLC43A2‐like), in pyloric caeca, midgut, and hindgut of triploid and diploid rainbow trout using RT‐qPCR. Housekeeping gene EF*α*1 was used for normalization of mRNA abundance data. Values were expressed as means ± SEM (*n* = 8–10). Asterisks represent significant differences between ploidies (Student’s *t*‐test, *P* < 0.05).

B^0^AT1‐like (SLC6A19‐like) is a Na^+^‐dependent, low‐affinity apical transporter that found to be expressed throughout the trout gut. Its mRNA expression was significantly higher in diploid than in triploid (Fig. 7, *P* = 0.009 and *P* = 0.01 in MG and HG, respectively, Student’s *t*‐test) associating with the observed Met flux across the mucosa at mmol/L concentration. Whereas, the low affinity sodium independent transporter mRNA expression of LAT3 with its undetermined location was low and there was no significant difference between triploid and diploid, exception for PC (Fig. 8A). On the other hand, LAT4‐like (SLC43A2‐like) is a Na^+^‐independent, low‐affinity transporter which typically is located in basolateral membrane. Its mRNA expression tended to be higher in 2N compared to 3N fish (Fig. 8B), again supporting greater flux across the serosa. That being said LAT3 contribution is likely minor compared to B^0^AT1‐like due to the lack of Met transport in sodium‐independent experiments along with the low expression of LAT3 mRNA. Thus, B^0^AT1‐like is likely to be the major contributor to apical transporter mediating Met flux across the intestine at mmol/L concentration. Whereas, transporting Met from enterocytes to the basolateral side is most likely controlled by LAT4‐like.

#### Electrophysiological recordings at mmol/L concentration

Attempt to characterize electrogenics at mmol/L concentration were inconclusive (data not shown), with electrophysiological changes not fitting kinetic models. This is likely due to the competing or opposing electrical signals generated by the apical and basolateral sodium dependent transporters that are more closely paired at mmol/L concentration.

## Discussion

Despite its important roles, there is a scarcity of research investigating the mechanisms of Met absorption in the fish intestine. This study was conducted to differentiate transport routes and transporters participating in Met transport in the trout gut. Here, we postulate that ASCT2 and y^+^LAT1 are responsible for Met uptake at *µ*mol/L concentration into the enterocyte from the apical and basolateral locations, respectively. There are five lines of evidence to support our assumption: (1) lack of Na^+^‐independent kinetics, (2) low expression of Na^+^‐dependent B^0^AT2‐like gene, (3) Na^+^‐dependent, high‐affinity (*K*_m_, *µ*mol/L ranges) Met radiotracer flux, (4) association of ASCT2 and y^+^LAT1 mRNA expression with high‐affinity kinetics, and (5) negative electrogenic currents in PC & MG, positive electrogenic currents in HG induced by Met. Whereas, B^0^AT1‐like is responsible for absorption at mmol/L concentration based on the gene expression and Na^+^‐dependent, low‐affinity kinetics (*K*_m_, mmol/L ranges). Finally, it would appear that major route of exit for Met from the basolateral side of the epithelium is through LAT4.

### **ASCT2 and y^+^LAT1 associated with DL‐Met transport at concentration gradient 0‐150 *µ*mol**/L

#### Absences of Na^+^‐ independent transport kinetics support ASCT2 and y^+^LAT1 function

At the concentration gradient 0–150 *µ*mol/L, DL‐Met transport strictly depends on the presence of Na^+^. Removal of Na^+^ from the buffer abolished all kinetics properties of DL‐[^14^C] Met flux. This is consistent with the absence of b^0,+^AT, the Na^+^‐independent high‐affinity transporter, in the genome, despite the presence of mRNA expression for rBAT‐ the heavy nonconducting subunit of b^0,+^AT. Although there is no saturable kinetics observed in our flux study in Na^+^‐free buffer, we cannot rule out the possibility that rBAT/b^0,+^AT might act as an exchanger exporting Met back into the lumen once Met has been accumulated inside the enterocytes. This assumption is supported in several studies suggesting that rBAT/b^0,+^AT typically behaves as an obligatory antiporter facilitating the influx of cationic AAs at the expense of neutral AAs efflux (Busch et al. 1994; Chillarón et al. 1996; Pfeiffer et al. 1999; Torras‐Llort et al. 2001). However, the role of rBAT in this study is not fully interpreted as there was no genomic presence of b^0,+^AT to create primers for expression analysis. The expression of rBAT without b^0,+^AT is odd, suggesting rBAT interaction with other transporters in fish species. Nonetheless, no Na^+^‐independent mucosal to serosal flux was observed at *μ*mol/L concentration ruling out a significant contribution of rBAT/b^0,+^AT. Therefore, absences of Na^+^‐ independent transport kinetics and gene expression support ASCT2 and y^+^LAT1 function in Met transport.

#### Low expression of Na^+^‐dependent B^0^AT2‐like gene support ASCT2 and y^+^LAT1 function

The question arising is that which Na^+^‐dependent, high‐affinity transporter is responsible for Met‐uptake at the concentration gradient 0–150 *µ*mol/L. RT‐qPCR results revealed three important gene candidates B^0^AT2‐like, ASCT2, and y^+^LAT1 which could explain the high‐affinity sodium‐dependent flux. However, B^0^AT2‐like mRNA expression was relatively insignificant (Fig. 3B), particularly in the diploid fish compared to ASCT2 and y^+^LAT1. Hence, it may not directly contribute to the kinetics observed in diploid and only minor contribution in triploid. This leaves ASCT2 and y^+^LAT1 as the best‐fit Na^+^‐dependent, high‐affinity candidates for transporting Met at the concentration of 0–150 *µ*mol/L.

#### Na^+^‐dependent, high‐affinity (*K*_m_, *µ*mol/L ranges) Met radiotracer flux support ASCT2 function

Observations in the current study predict that DL‐Met transport is mediated by a Na^+^‐dependent, high‐affinity transporter ASCT2. In Na^+^ conditions, measurement of flux rate versus substrate concentration displayed typical Michaelis–Menten curves with high affinity (*K*_m_ between 4 and 5 *µ*mol/L). These values are lower than *K*_m_ values generated by ASCT2 in other research such as 240 *µ*mol/L in pregnant mice tissues (Verma and Kansal 1993), and 288 *µ*mol/L in mouse testis (Utsunomiya‐Tate et al. 1996). The gap in the affinity of the current research with previous studies could be due to two reasons. Firstly, different research techniques are carried out. In our study, tissues were used, and the experiments were performed at a cold physiological temperature of trout (12^0^ C); while vesicles, cell lines, or transporter‐expressed *Xenopus oocyte* were carried out at room temperature or warmer. Secondly, kinetic properties of a transporter could differ from one species to another to some extents. For example, the *K*_m_ for D‐Glucose transported by GLUT1 (SLC2A1) varies greatly within species and taxonomic lines; namely 2.5–75 mmol/L in human (Burant and Bell 1992; Day et al. 2013; Gould et al. 1991; Hahn et al. 1998; Woodrow et al. 2000), 14–26 mmol/L in rat (Nishimura et al. 1993; Regina et al. 1997), or 9.3 mmol/L in rainbow trout (Teerijoki et al. 2001).

#### Gene expression support ASCT2

In the flux studies, *K*_m_ values are similar along the intestine while *J*_max_ in PC, MG, and HG of 3N is statistically higher than 2N trout. A similar *K*_m_ implies that Met is likely mediated by the same type of transporter along the entire intestine. Meanwhile the difference in *J*_max_ suggests different density of transporter expression. This correlates well with RT‐qPCR results (Fig. 3A) where higher ASCT2 mRNA expression in the 3N fish is observed, compared to that of 2N fish (*P* = 0.005 and 0.026 in PC and MG respectively).

#### Negative currents in PC & MG, and positive currents in HG induced by DL‐Met support ASCT2 and y^+^LAT1 functional contribution

The functional contribution of ASCT2 and y^+^LAT1 was further affirmed by electrophysiological recording associated with the expression of these two transporters. In Na^+^‐containing buffer, robust negative *I*_sc_ in PC and MG and positive *I*_sc_ in HG in both 2N and 3N were found when increasing the concentration of DL‐Met.

To give clarity, in our Ussing chambers a negative current would occur if an anion is moving in the mucosal to serosal direction or a cation is moving in the serosal to mucosal direction. On the other hand, a positive current would occur if a cation is moving in mucosal to serosal direction or an anion moving from the serosal to mucosal direction. With this above in mind, it appears that the negative current in PC and MG is due to Met transport through basolateral y^+^LAT1 with sodium. It is widely reported that y^+^LAT1 is an AA exchanger that mediates influx of basic/cationic AAs within the enterocytes into the blood and re‐influx of neutral AAs from the blood back into the enterocytes (Bröer and Fairweather 2019; Pfeiffer et al. 1999). It transports basic AAs without the requirement of Na^+^, whereas it is dependent on Na^+^ when transporting neutral AAs (Fotiadis et al. 2013; Kanai et al. 2000; Torrents et al. 1998). This process of neutral AA absorption is thought to be generally electroneutral exchanging the available intracellular basic amino acids for Na^+^ and the neutral AA. However, in our Ussing chamber assay we did not supply cationic amino acids. Thus, this created a very limited intracellular pool of cationic amino acid to exchange with Met and Na^+^. This would result in an electrogenic signal from y^+^LAT1 as Met, a neutral AA can stimulate the efflux of neutral AA through y^+^LAT1, while also driving Na^+^ uptake (Kanai et al. 2000). Thus, the negative currents observed here are likely due to Na^+^ and accumulated fluxed Met entering from the basolateral side of the enterocytes via y^+^LAT1. This electrical contribution from y^+^LAT1 may also explain why interpretable electrophysiological data was unattainable at mmol/L concentration, where B^0^AT1‐like electrical signal (apical entry of sodium) would be masked by y^+^LAT1 electrical signal (basolateral sodium entry in the absence of cationic amino acids).

On the other hand, the positive currents in the HG is likely due to the dominate expression of ASCT2 on the apical membrane and a decrease in y^+^LAT1. Although ASCT2 is generally reviewed as a transporter transporting alanine, serine, cystine, glutamine, and asparagine at high affinity (Pinho et al. 2007; Scopelliti et al. 2018), it also accepts methionine, leucine, and glycine with lower affinity (Pingitore et al. 2013; Utsunomiya‐Tate et al. 1996; Verma and Kansal 1993). Studies on the functional characteristics of ASCT2 confirms that the transporter is an obligatory antiporter (exchanger) which utilizes Na^+^‐electrochemical gradient to equilibrate cytoplasmic glutamine and neutral AAs pools, but the exact stoichiometry of the transport is still unknown (Pingitore et al. 2013). The matter of net electrical ion flux generated by ASCT2 is a subject of controversy (Broer et al. 1999; Scalise et al. 2014; Utsunomiya‐Tate et al. 1996). That means that the positive current in the hind gut is likely not due to sodium transport by ASCT2. However, ASCT2 has also been characterized to have channel like anion conductance (Broer et al. 2000). Thus, if ASCT2 anion conductance is activated by amino acid transport, the resting membrane potential of the epithelial cell would likely drive chloride out of the cell into the lumen creating a positive current. More specifically, the reduction of basolateral y^+^LAT1 would reduce sodium entry on the basolateral membrane (a serosal to mucosal movement) contributing less negative current to the overall tissue and the increase of apical ASCT2 would result in more chloride movement out the cell to the mucosal surface creating a positive current.

Additionally, the higher *V*_max_ in the 3N was associated with higher expression of ASCT2 in the PC and MG. The higher ASCT2 likely accounts for the larger mucosal to serosal flux, which would then result in higher basolateral methionine concentration available for y^+^LAT1 reabsorption causing a larger negative *V*_max_ in triploid (Fig. 3A). In short, the dominant functional y^+^LAT1 expression would produce a negative current when bringing sodium and methionine into the cell on the basolateral side of the epithelium. This is further supported by gene expression and the positive currents in the HG, where expression of ASCT2 is much higher than the expression y^+^LAT1.

The low expression of the sodium dependent transporter B^0^AT2‐like, and poor association with changes in *I*_sc_ suggests a very minor contribution in 3N and almost none in 2N. Therefore, B^0^AT2‐like is unlikely to account for the robust changes in *I*_sc_ observed between segment and ploidy. Hence, ASCT2 and y^+^LAT1 are the most likely major contributors to mediating DL‐Met electrogenic currents at the concentration range of 0–150 *µ*mol/L.

### **B^0^AT1‐like transporter associated with DL‐Met influx at concentration gradient 0.2‐20 mmol**/L

At the concentration gradient 0.2–20 mmol/L, DL‐Met transport was also dependent on Na^+^ as the driving force for absorption. Repeatedly, the flux of DL‐[^14^C]Met was absent when sodium was removed from the buffer. Flux measurement without Na^+^ could not be mathematically modeled using both linear and nonlinear regression. This reinforces the role of sodium in regulating AAs absorption of teleost species (Balocco et al. 1993; Ingham and Arme 1977; Lucia et al. 2001; Maffia et al. 1990; Mepham and Smith 1966; Miller and Kinter, 1979; Smith 1969; Vilella et al. 1988).

Supporting this sodium dependent flux at mmol/L concentrations was the strong expression of B^0^AT1‐like transporter gene. B^0^AT1, is a Na^+^‐dependent apical transporter, typically has a low affinity (*K*_m_ in mmol/L ranges) likely eliminating its contribution to the flux at *μ*mol/L concentration (Bröer 2008; Kleta et al. 2004). Therefore, the B^0^AT1‐like transporter is the sole candidate gene that could participate in Met absorption at high substrate concentration. This is supported by the gene expression which is higher in diploid compared to triploid (Fig. 7), resulting in greater *J*_max_ in diploid. The transporter belongs to system B^0^. The system has been previously identified in brush border membrane preparations (Munck and Munck 1994; Preston et al. 1974), a bovine renal epithelial cell line (Doyle and McGivan 1992), and Caco‐2 cells (Souba et al. 1992). These works have demonstrated that system B^0^ accepts all neutral AAs, obviously with varying affinity levels. Typically, long side chains such as methionine, phenylalanine, and leucine are transported with higher affinity (*K*_m_ ranging from 1.5 to 4 mmol/L) than other neutral AAs such as alanine and glycine (*K*_m_ > 15 mmol/L). Preston and coworkers (1974) found that the L‐Met maximal influx and affinity in rabbit ileum were 2.2 *µ*mol/cm^2^·h and 1.6 mmol/L, respectively, over a concentration range of 0.1–16 mmol/L. This is relatively compatible with our study in terms of technique and results. The *K*_m_ calculated in the present study for high concentration 0.2–20 mmol/L were between 0.6 and 1.0 mmol/L in both triploid and diploid trout (Table 4). The maximal flux in our study is lower, which could be due to differences in species, the isomeric form of substrate, or the involvement of other Met transporters.

#### Lower *J*_max_ at mmol/L concentration

Noticeably, the *J*_max_ observed in the mmol/L concentration gradient is lower than the *J*_max_ in the *μ*mol/L concentration gradient. A comprehensive explanation for this phenomenon requires understandings about transport modes and expression of genes involved. Met exits from the enterocytes across the basolateral membrane is most likely mediated by LAT4‐like transporter, based on expression levels (Fig. 8B), the absence of other known basolateral transporters in the genome and LAT4’s previously defined basolateral function in mammalian species. The preferential substrates of LAT4 included branched‐chain AAs, phenylalanine, and methionine (Bodoy et al. 2005). Transport via LAT4 expressed in *X. laevis oocytes* is Na^+^‐independent with low affinity: *K*_m_ for L‐phenylalanine was between 4 and 6 mmol/L (Bodoy et al. 2005; Guetg et al. 2015). The role of LAT4 was further validated with a knockout model in which mice lacking LAT4 protein suffered growth defects and early postnatal lethality, presumably due to malnutrition with low Met and branched‐AAs in plasma (Guetg et al. 2015).

However, the expression of the LAT4‐like transporter is substantially less than that of B^0^AT1‐like transporter potentially resulting in DL‐Met accumulation in the enterocytes. If this is occurring, accumulative cytosol Met must be exported back into the lumen. This may explain why the maximal flux rate is smaller in HG than in PC and MG, due to high expression of ASCT2. This notion is supported by the observation that several AAs can be bidirectionally transported by ASCT2 (Broer et al. 1999; Deitmer et al. 2003). For example, human ASCT2 was expressed in *Pichia pastoris* showed that extracellular side displays high affinity (micromolar range *K*_m_), while intracellular side shows low affinity (millimolar range *K*_m_) when analyzing the kinetics of [^3^H]glutamine (Pingitore et al. 2013). Thus, in our study when methionine reaches mmol/L concentration, ASCT2’s low internal affinity could then transport methionine back to the lumen decreasing *J*_max_. However, based on our current understanding of ASCT2 stoichiometry it likely only recycles at the higher concentration. Thus, this suggests an alternative channel with an internally low *K*_m_ allowing back flux. A possible candidate would be b^0,+^AT as we have found high expression of associated rBAT (data not shown). However, we were unable to find b^0,+^AT in the trout genome, suggesting that a yet to be identified channel interacts with rBAT. This is not surprising as rBAT has been suggested to associate with an unidentified subunit in the kidney (Fernández et al. 2002).

### Limitations of the study

The techniques and experiments used in this study have a couple of limitations. Firstly, mRNA levels are not necessarily correlated with protein expression. However, currently there are no available fish antibodies for these transporters to be assessed by Western blot. Secondly, there is a potential for the existence of unique paralogs for each transporter due to the two whole genome duplication events that trout undergone. Finally, the use of inhibitors would have been advantageous to confirm the role of the associated transporters involved. However, we have found that mammalian inhibitors generally do not work in trout. We suspect that this is due to the lower temperature at which the experiments are run**.** Our previous work demonstrated that glucose transport inhibitors work in tilapia at 26°C but not in trout at 12°C (Subramaniam et al. 2019). This is not too surprising as temperature is known to affect a drugs ability to inhibit its target (Forsling 1968). Unfortunately elevating the temperature of the trout tissue resulted in death of the tissue.

## Conclusion

**Figure 9:**
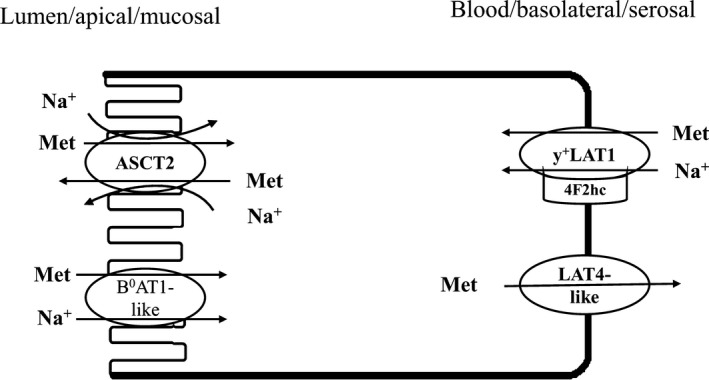
Schematic model of Methionine transport mechanism in the gastrointestinal tract of rainbow trout. At *µ*mol/L concentration, Met uptakes across the mucosal membrane of epithelial cells are facilitated by the Na^+^‐dependent high‐affinity apical transporter ASCT2 (SLC1A5). At mmol/L concentration, Met is taken up into the epithelium via the Na^+^‐dependent low‐affinity apical transporter B^0^AT1‐like (SLC6A19‐like). Whereas, the basolateral transporter LAT4‐like (SLC43A2) appears to be the sole gate controlling Met exit from enterocytes into the blood stream. Presence of 4F2hc/y^+^LAT1 (SLC3A2/SLC7A7) on the serosal side allows some Met to be recycled back into enterocytes, and eventually exported back into the lumen via intracellular low affinity ASCT2.

Amino acid transporters have been intensively studied in the intestine of mammalian species. Here we present the very first description of the kinetic properties of Met epithelial transport and associated transporter gene expression in fish intestine. Similar to mammals, sodium is physiologically important for many nutrient transport function in fish. In this study we have clearly demonstrated that it is the driving force governing Met absorption in trout intestine through the Na^+^ dependent transporters. The mucosal to serosal flux of DL‐Met at *µ*mol/L concentration gradient seems to be primarily governed by the apical ASCT2 transporter. This was supported by gene expression, Na^+^‐dependence, and a high affinity kinetics (*K*_m_ in *µ*mol/L ranges) that are relatively similar to basic functional properties of ASCT2 that have been described by others in literature (Broer et al. 2000; Gliddon et al. 2009; Scalise et al. 2014). Meanwhile y^+^LAT1 may play a role in the basolateral reabsorption of methionine. This is supported by electrogenics and gene expression of y^+^LAT1. At mmol/L concentration, evidence including Na^+^‐dependence, low affinity kinetics (*K*_m_ in mmol/L ranges), and association of gene expression suggest that transport at these concentrations is primarily mediated by apical B^0^AT1‐like transporter. Genomic and gene expression analysis along with lower *J*_max_ in the mmol/L concentration suggest the sole contribution of basolateral exit of methionine through a LAT4‐like transporter. This first description begins to define the overall mechanism of methionine transport in trout intestine which is summarized in Figure 9.

## Conflict of Interest

The authors declare that there are no conflicts of interest, financial or otherwise regarding the publication of this paper.
